# Understanding the application of handwritten text recognition technology in heritage contexts: a systematic review of Transkribus in published research

**DOI:** 10.1007/s10502-022-09397-0

**Published:** 2022-06-17

**Authors:** Joe Nockels, Paul Gooding, Sarah Ames, Melissa Terras

**Affiliations:** 1 School of Literatures, Languages and Cultures, University of Edinburgh, Edinburgh, Scotland; 2 Information Studies, College of Arts, University of Glasgow, Glasgow, Scotland; 3 National Library of Scotland, Edinburgh, Scotland; 4 College of Arts, Humanities and Social Sciences, University of Edinburgh, Edinburgh, Scotland

**Keywords:** Digitisation, Digital library, Handwritten text recognition, Systematic literature review, Transkribus, Artificial intelligence

## Abstract

Handwritten Text Recognition (HTR) technology is now a mature machine learning tool, becoming integrated in the digitisation processes of libraries and archives, speeding up the transcription of primary sources and facilitating full text searching and analysis of historic texts at scale. However, research into how HTR is changing our information environment is scant. This paper presents a systematic literature review regarding how researchers are using one particular HTR platform, Transkribus, to indicate the domains where HTR is applied, the approach taken, and how the technology is understood. 381 papers from 2015 to 2020 were gathered from Google Scholar, Scopus, and Web of Science, then grouped and coded into categories using quantitative and qualitative approaches. Published research that mentions Transkribus is international and rapidly growing. Transkribus features primarily in archival and library science publications, while a long tail of broad and eclectic disciplines, including history, computer science, citizen science, law and education, demonstrate the wider applicability of the tool. The most common paper categories were *humanities applications* (67%), *technological (25%), users* (5%) and *tutorials (3%)*. This paper presents the first overarching review of HTR as featured in published research, while also elucidating how HTR is affecting the information environment.

## Introduction

Handwritten text recognition (HTR) technology is now a mature machine learning tool, capable of producing accurate machine processable text from images of historical manuscripts. As a result, it has been used by libraries and archives, speeding up the transcription of primary sources and facilitating full text searching and analysis of historic texts at scale. However, there is very little published research that considers how this technology is being applied or utilised by institutions or researchers. We present a comprehensive view of how HTR is mentioned in published research, via a systematic literature review focussing on the Transkribus platform. Transkribus is the most popular user-facing platform for producing transcripts of historical texts across the cultural and heritage industries (READ 2021). As such, Transkribus is the sole focus of this paper. However, a brief synopsis of how HTR technology was developed, and an overview of other HTR providers, is given. This state of the field assessment analyses how HTR is being deployed, used, and reported in published research.

Transkribus allows for the automated recognition and transcription of historical texts, making these materials more readable and, in turn, broadening access to collections and extending understandings of the past (Muehlberger et al. 2019). Transkribus originated from an EU FP7 funded project ‘Transcriptorium’, and then from an EU Horizon 2020 funded project, READ (Recognition and Enrichment of Archival Documents), which launched an online HTR tool in 2015. It has since been developed further by the READ-COOP, structured around a cooperative of partner institutions and becoming a paid-for service in 2020. As the heritage sector becomes more dependent on digitisation, Transkribus acts (alongside other services) as an accessible tool for institutions, tying into ideas of ‘Collections As Data’ as a computational way of making historical collections more accessible and processable (Lincoln 2017). Currently, Transkribus is the most commonly used HTR tool in the cultural heritage space, with around 1700 regular monthly users. As such, Transkribus is an ideal focus of study in understandinghow HTR (as a mature instantiation of machine learning) is being used by heritage institutions.

HTR is closely related to older Optical Character Recognition (OCR) technology, which was initially developed to focus on the accurate identification of single characters in predictable printed text. OCR became widely available in the 1990s through ABBYY producing its first popular product in 1993 (ABBYY 2021 https://www.abbyy.com). However, off-the-shelf OCR software presented issues of limited customisability, with problems including the expense of page-limits for smaller repositories, limited layout analysis options in later iterations of tools such as ABBYY Finereader 9.0 CLI, and poor effectiveness on complex or changeable scripts such as handwriting (Blanke et al. 2012). That said, OCR is still widely utilised, after having been dramatically improved with the use of Hidden Markov Models (HMMs), a family of tools modelling sequential processes first used in speech recognition, utilised broadly in the pre-processing stages and text recognition stages (Impedovo 1993). There are now various OCR tools in routine use, including Adobe Acrobat, which began supporting ClearScan from 2008, and the conversion of scanned images of print to machine-readable text from 2012 with Adobe Acrobat Pro DC (Adobe 2021 https://www.adobe.com/uk). In recent years, OCR has integrated machine learning techniques, improving accuracy rates, but characters still must be isolated and spatially separated and complex layouts, fonts and media can cause poor results (Cordell 2017). That said, Tesseract, a major commercial OCR package, is utilised for a variety of means, from text detection on mobile devices to deciding whether an email is spam or not based on its content (Tesseract 2021 https://github.com/tesseract-ocr/tesseract). While less established, the open source OCR programme Kraken has also produced similar outputs to Tesseract while embedding pre-processing steps within the software, such as the binarization of document images to aid in recognition (Kraken 2021 https://medium.com/analytics-vidhya/unleashing-the-kraken-for-ocr-fba6bff73c8c). The move from OCR to more advanced HTR, which uses machine learning approaches such as deep neural networks to extract visual features and recognize characters and words in a segmented line of text via the calculation of overlapping probabilities, has brought noticeable improvements. These are especially evident in the accurate line segmentation and the decipherment of more complicated glyphs (Edwards 2007). Like its OCR counterparts, HTR requires some manual intervention and training, yet lessens the need for full human transcription and bespoke recognition models developed at high cost.

There are a variety of HTR tools at researchers’ disposal, which can improve collection description, information retrieval and recognition of historical documentation. Many projects have developed their own bespoke HTR solutions, working in interdisciplinary teams with computing scientists (Terras 2021). A tool called Monk, developed by Lambert Schomaker at the University of Groningen, looks to help scholars in writer identification and style-based dating (Monk 2004 https://www.ai.rug.nl/~lambert/Monk-). Labels are added at the description level, along with line transcriptions and broader zone labels for words, allowing a scholar to create a growing index of documentation (Schomaker 2020, pp. 226-227). Since Monk went live in 2009, the corpus of materials ingested into the system has grown greatly, starting with fifteenth century texts and, in 2014 and 2015, respectively, moving onto processing Chinese and Arabic characters. The most recent figures for the total number of harvested and human-confirmed word labels stood, as of 2013, at around 370,000 (Monk 2004 https://www.ai.rug.nl/~lambert/Monk-). While complementary work should be undertaken on how Monk is mentioned in research, this article will surface how the major user-facing HTR platform for historical documents, Transkribus, is used by the research community. The scale and scope of Transkribus, including its growth in users, and their resulting community of practice (Wenger 1999), will present an insight into how HTR is being deployed, utilized, and reported in published research. Furthermore, this paper establishes a methodology for comparing discussions and the usage of HTR platforms more broadly, which could aid future research looking at disciplinary differences in tool adoption.

## Methodology

Previously published research mentioning Transkribus (a unique string making identification easy) was collated using refined and restricted searches in Google Scholar (GS) (2004), Scopus (2004), and Web of Science (WoS) (2020), following a methodology used to analyse microblogging research on Twitter by Williams et al. (2013). The three databases are distinct tools which provide complementary benefits, given no database has universal coverage. Scopus is the largest database of peer-reviewed literature and provides analytical tools to ensure errors of omission do not occur. GS has extensive coverage of grey material and non-scholarly sources (such as promotional pages, tables of contents and course reading lists), allowing the review to move beyond the main journals. WoS proved helpful in finding academic materials missed by GS, querying both science and arts and humanities indexes. This cross-platform search allowed for the checking of catalogue entries’ semantic validity and relevance. Item metadata was exported from GS, Scopus and WoS to the referencing software Zotero (2006). At this stage, metadata records were cleaned, enhanced with missing data, and checked for duplicate results, before being ordered. The corpus was interrogated using a blend of quantitative content analysis and qualitative methods, coding the unstructured data around certain themes (Berelson 1952; Drisko and Maschi 2015) and grouping materials by typology (journal, review, tutorial) and theme.

Four overarching themes were identified through a close reading of the tonal vocabulary of abstracts, keywords, and titles: *humanities application*, *technological*, *tutorial* and *user*. A limitation of this research is the minimal description of method, subject, approach and findings found in abstracts (Weber 1990). Without a full reading of all the entries in this literature review, ascertaining the precise nature of results is difficult. Nevertheless, this multistage approach allowed subunits of interest to emerge, as well as a replicable process of data analysis from multiple vantage points (Krippendorff 2004). In turn, this method indicated how researchers advertise their contributions to the Transkribus literature; present their findings; what approaches they prize above others; and how the HTR tool is used across humanities disciplines. The metadata was exported to Excel for data analysis and visualisation. New ways of viewing the dataset continued to be developed, identifying gaps until saturation was reached (Charmaz 2006). Macroscale and microscale approaches are therefore brought together as complimentary methods, from which written and numerical conclusions can be drawn (Jockers 2013).

### About the corpus

**Fig. 1:**
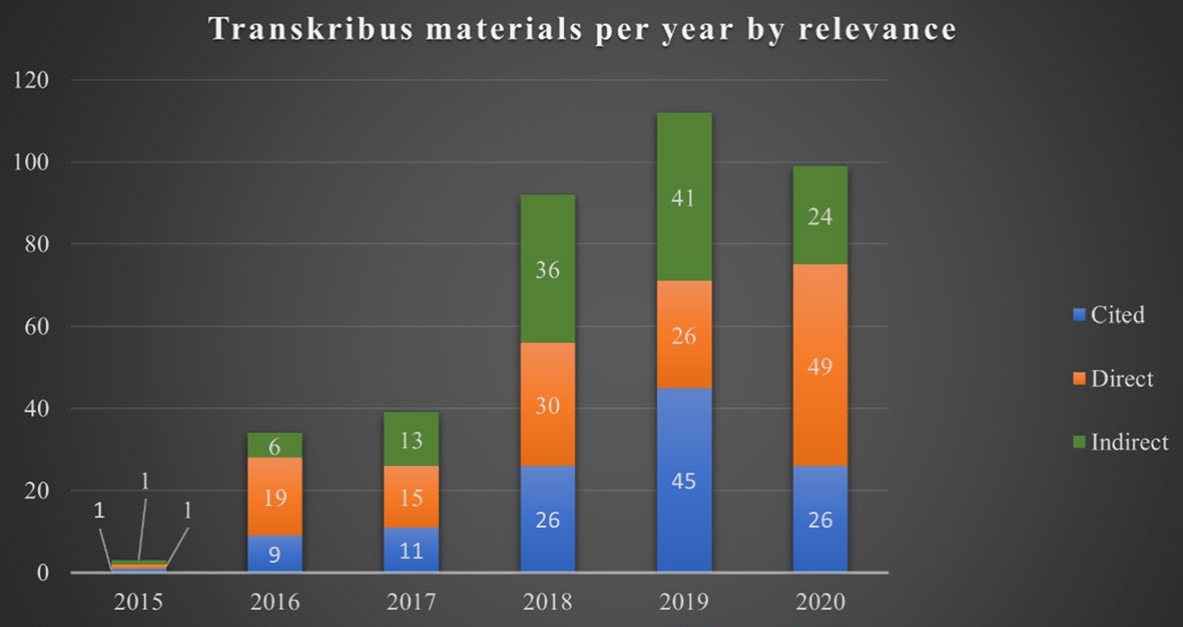
Chart showing the gathered Transkribus publication corpus by year and relevance

381 publications were catalogued in total. 85% of results came solely from GS, 1% solely from Scopus and 11.3% from WoS. The small percentage of entries returned by Scopus can be accounted for as another 2.6% of results were found in two or three of the databases: producing a large amount of duplicates. There has been a steady rise in Transkribus research, as shown in Fig. 1 below, since 2015, when there were 3 publications returned: rising to 34 recorded results in 2016. The most notable rise occurred between 2017 and 2018: 2017 returned 39 works, whereas, in 2018, 92 materials were recorded. With 112 works, 2019 saw another increase in scholarly materials mentioning Transkribus. 2 recorded items are not factored into this chart, as GS returned 1 outlier paper from 2010 and another was undated.

99 results were gathered from 2020, despite the indexing for the review finishing in late October of that year (and potential delays in publication caused by the Covid-19 pandemic). HTR’s rise in mentions shows a distinct shift in the collection and curational landscape, with memory institutions opting for a greater digitisation of their materials (Chassanoff 2013; Duff et al. 2004). Journal articles were the dominant format of materials, accounting for 42.78% (*n* = 163) of the full dataset, 21.00% (*n* = 80) were conference papers; 8.14% policy documents (*n* = 31), such as those from READ; 7.61% book sections (*n* = 29); 6.56% theses (both undergraduate and postgraduate) (*n* = 25); 4.99% reports (*n* = 19); and 4.72% presentations (*n* = 18). The remaining 4.20% (*n* = 16) were a mix of blog posts, magazine articles and video recordings, mostly tutorials of the Transkribus platform. If we are to just take those works directly featuring Transkribus, the split remains similar: 52.14% of publications were journal articles (*n* = 73) and 17.14% (*n* = 24) were conference papers. The Transkribus research indexed is clearly weighted towards academic formats, due to the nature of the databases used. However, it should be considered that discussions are likely also occurring in informal publications such as blog posts and forums.

Out of the entire dataset of 381 works, 71.13% of works were accessible online under an openly accessible license (*n* = 271). Out of these, 36 were located in institutional repositories, and 235 were freely accessible online, including articles (*n* = 113), conference papers (*n* = 33) and presentations (*n* = 15). Works which were not regarded as open access made up 22.31% of the corpus (*n* = 85), requiring access to subscription resources. Most of these works were conference papers (*n* = 42) and journal articles (*n* = 30), and book sections or monographs (*n* = 14). 3.67% of materials were published titles which varied in terms of accessibility (*n* = 14) and 3.89% (*n* = 11) could not be determined. The fact that the majority of the literature is available for those in the scholarly community holds similarities with the collegial nature of the READ-COOP, analysed further below, while the range of publication venues shows the active use of and scholarly discussions mentioning the platform.

## Corpus stratification

Transkribus was mentioned in publications to varying degrees, necessitating the recording of how each source engaged with the platform. 31.97% of the corpus (*n* = 118) merely ‘cited’ Transkribus: either via direct citation or a demonstrable impact upon the researcher’s approach without explicit inclusion in the main text. These works predominantly came from GS (95.76%) or WoS results (2.54%), with 1 result appearing in both GS and WoS (0.85%), and another in GS and Scopus (0.85%). 32.28% of the dataset (*n* = 123) related to materials which were ‘indirectly’ focused on Transkribus, discussing it explicitly, often at length, but not as a focal point of discussion. This was mainly seen in articles where Transkribus’s capabilities were compared to another platform, such as Tesseract or ABBYY FineReader. Most commonly, 36.75% (*n* = 140) of papers ‘directly’ mentioned Transkribus as their primary subject. Many of these texts were written by researchers who had used the software themselves (Romein 2019). Other works provided tutorials or considered the future possibilities of Transkribus once it gained greater accuracy in its outputs. Therefore, the findings presented here utilise two different samples: the entire corpus of Transkribus material, including ‘cited’ works; and those from materials directly focused on the HTR software.

## Findings

### Internationalisation

**Fig. 2:**
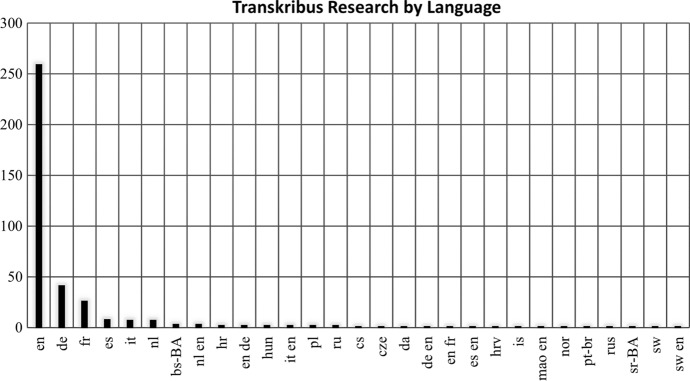
Chart showing the distribution of Transkribus research by publication language (given in language code). Some entries are charted as having two languages, e.g. ‘nl en’ for Dutch-English. These works were published with translations. Both languages have been recorded, not just the original, to gain a more accurate sense of Transkribus research’s publication context

67.98% of Transkribus publications were written in English (*n* = 259) (for journal articles that directly mentioned Transkribus, the percentage in English becomes higher at 85.00%, *n* = 119), see Fig. 2, with the next most prevalent language being German at 10.76% of results (*n* = 41). Nevertheless, across all 381 papers we discovered, Transkribus research appears more multilingual than science and humanities databases generally. The percentage of English language content in Scopus reached 88.4% in 2013, with 77% of Arts & Humanities materials appearing in English (Van Weijen 2013). More recent studies have shown that the prevalence of Standard English is on the rise, irrespective of field, estimating that 98% of publications in science are written in the English language, causing researchers from English as a Foreign Language (EFL) countries to sound the alarm that their contributions are being inhibited (Flowerdew 2013; Ramirez-Castaneda 2020). However, this does not appear to be the case with Transkribus research.

Among the English language items, 37.84% appeared as journal article contributions, in comparison to 51.22% of German language materials, reflecting Transkribus’ international use, setup and development. 6.82% of materials were written in French (*n* = 26), notably as reports and scholarly articles: such as the work of Massot et al. (2018) in disseminating transcribed reading sets of Foucault’s writing preparations. English, while being the  most common language of Transkribus research, sits alongside results in Dutch, Spanish; Swedish, Bosnian, Russian, Norwegian, Polish, Italian, Croatian, Hungarian, Czech and even Maori (the one example being a 2020 policy statement from New Zealand’s National Archive) providing a greater sense of the variety in cultural context among those researching and using Transkribus.

**Fig. 3:**
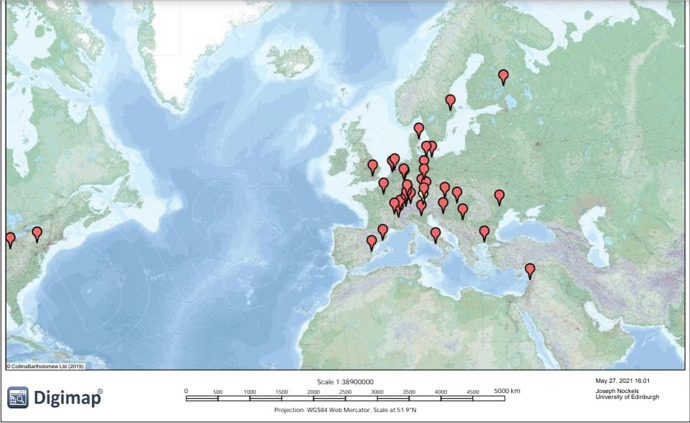
Direct Transkribus research (2015–2020) plotted using Digimap’s roam feature. Location markers identify the lead researchers’ affiliation. The Innsbruck server is located with a yellow pin

In order to analyse where outputs were published, we used Digimap (2020) and its global roam feature to plot entries based on the institutional affiliation of their lead author. As Fig. 3 shows, Transkribus is being used worldwide, although the European origins of the project are clearly reflected in the spread of publications. There is a strong German presence in the dataset. Materials from researchers stationed at the Universität Rostock (*n* = 6, 1.57%), which developed the CITlab basefinder used in the HTR engine for Transkribus, an initiative which made an impact in making the tool usable for a wider variety of researchers from 2018 onwards, as well as the Universitätsbibliothek Freiburg (*n* = 4, 1.05%), Friedrich-Alexander-Universität Erlangen-Nürnberg, Universität Greifswald and the Max-Planck Gesellschaft zur Förderung der Wissenschaften were all listed (*n* = 1 each, 0.26%). Transkribus and READ talk of ‘synergy, collaboration and the sharing of data and resources’, a goal which is being moved toward as the software gains momentum (READ 2020a, b). Nevertheless, *Stellenbosse Heemkring* (a local archive of the Cape Town suburb of Stellenbosch) is the only African institution to have signed a Memorandum of Understanding (MoU) as part of the 2016–2019 READ project, with no Asian or South American institutions yet involved. Individual researchers from the United Arab Emirates (UAE) and Kuwait are listed as members (receiving discounts, voting privileges at annual Transkribus conferences and added information about business policies) suggesting that Transkribus is gaining some traction in West Asia, a conclusion supported by Fig. 3 which shows Chammas’s work at the University of Balamand in Lebanon (2018). Despite that, concerns presented by affiliates of the READ-COOP SCE (European Cooperative Society) that HTR models being produced are stronger in terms of western languages (due to user bases being decidedly European), echo misgivings about English being the lingua franca of academic research (Researchers at University College Report 2019). Furthermore, Fig. 3 shows that research output is collected around the Transkribus server in Innsbruck (with a few exceptions mainly in Scandinavia, Eastern Europe, and the US). It remains hard to ascertain whether READ’s focus on enabling common transnational activities and benefiting user needs regardless of geographical location will change this paradigm, with much of the research into and using the platform remaining at Austrian and Germanic institutions.

Transkribus’s appeal as a flexible product (which was initially free to use) appears to be reflected in where research has occurred (such as the University of Belgrade and Moldova State University), rather than being expensive software used by a limited number of more affluent Western institutions. This may change, given the platform moved to a pay-for model in October 2020, which will be reflected in the future publication record and should therefore be monitored.

**Fig. 4:**
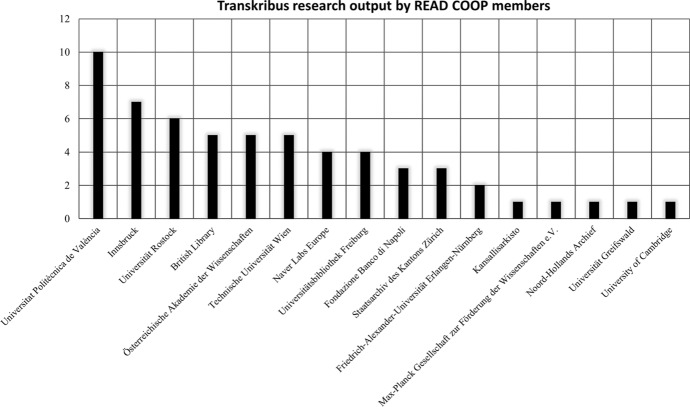
Chart highlighting the research output of institutional READ-COOP members by number of publications

Members of the READ-COOP have played a role in producing and publishing works related to Transkribus, with 30.97% (*n* = 118) of lead authors representing cooperative institutions. This percentage rises noticeably to 67.86% (*n* = 95) when focusing solely on works directly related to the HTR platform. In some cases, for example Schlagdenhauffen’s research (2020) using Transkribus to form semi-automated transcriptions of the diary of lawyer Eugène Wilhelm (1866–1951), work from the READ-COOP can be attributed to individuals joining the decision-making community independently of their institutions. Working from the READ-COOP membership roll found online (2020), 39.00% of READ-COOP members (*n* = 46) chose to register as anonymous members of the cooperative, limiting the information that could be gleaned about highly involved users of the platform from this data alone. That said, certain institutional members of the READ-COOP have been publishing research aligned with Transkribus: as shown in Fig. 4 below, 10 works (2.62%) came from the Universitat Politècnica de València, which has been instrumental in improving the recognition of document structure with its P2PaLA layout analysis tool, allowing the HTR to locate the linguistic components of historical materials accessible through natural language processing more thoroughly. 7 works (1.84%), mostly policy documents from READ, came from Innsbruck where the infrastructure of Transkribus is maintained.

READ-COOP members from outside Germany are also represented in the dataset, including several prominent cultural organisations: Kansallisarkisto (The National Archives of Finland) (*n* = 1, 0.26%), Naver Labs Europe based in France (*n* = 4, 1.05%), Fondazione Banco di Napoli (*n* = 3, 0.79%), and British institutions like the British Library (*n* = 5, 1.31%) and the University of Cambridge (*n* = 1, 0.26%). As with mapping open publishing onto the Transkribus dataset, it was not possible to identify whether 2.10% (*n* = 8) of the publications stemmed from READ-COOP members. These exceptions were mostly listed as 'cited' papers and were written by academics with no notable web presence, making categorisation difficult. It is clear, nonetheless, that the READ-COOP itself is becoming a strong space for likeminded institutions to utilise Transkribus and publish related research, increasing capabilities and presenting results to the research community.

### Themes and content

Of the 140 results mentioning Transkribus directly, only 10 examined OCR as an additional subject. Transkribus offered OCR capability, providing an inbuilt ABBYY Finereader function, before licensing issues in 2021. Out of the 10 indexed materials which mentioned OCR, only 2 abstracts included a description of using this function within Transkribus (Lindemann et al. 2018; Ströbel and Clematide 2019) while others used OCR externally through self-built platforms or those supplied by ABBYY (*n* = 2), suggesting that the licensing issue did not impact users greatly. Others mentioned OCR in comparison to HTR, comparing their accuracy rates in deciphering text (*n* = 6).

**Fig. 5:**
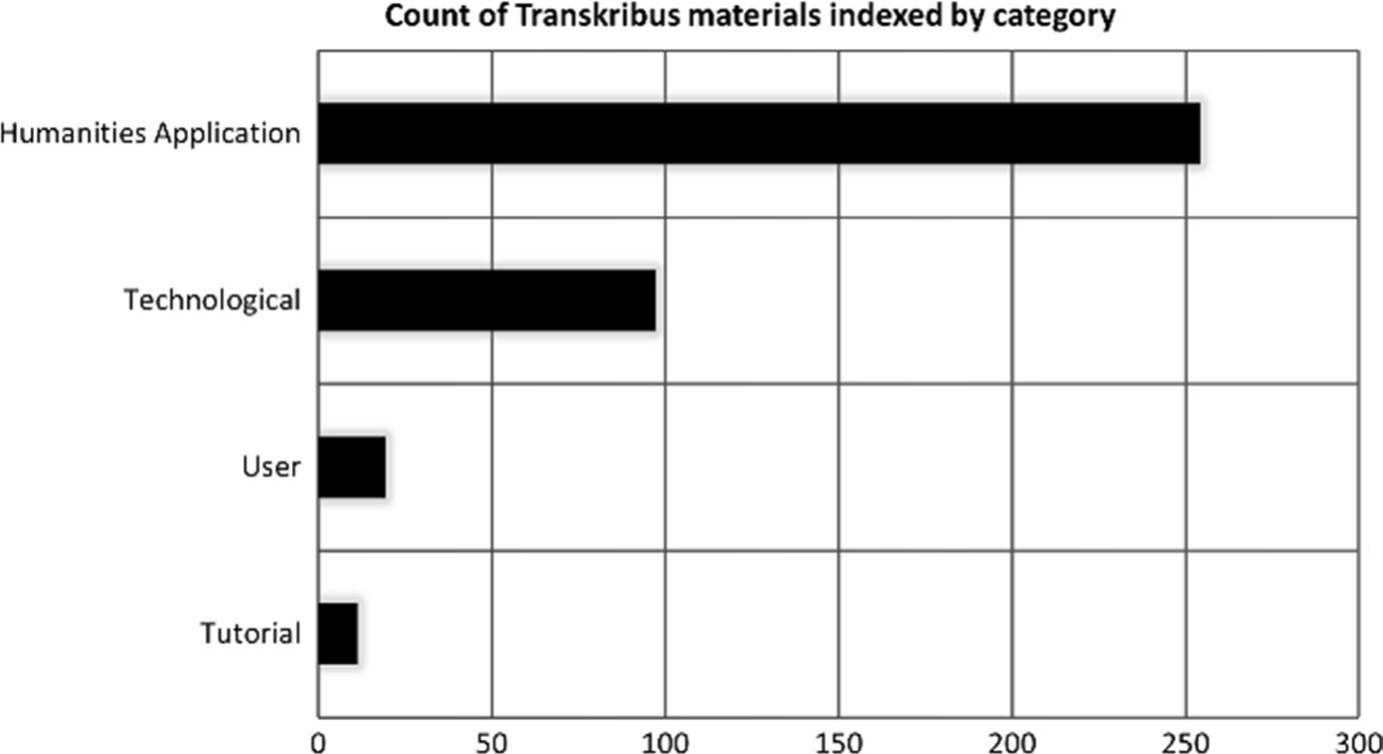
Chart detailing the breakdown of Transkribus materials by category

Papers in the corpus were categorised due to theme and essence. The *humanities application* category was defined as any material where researchers presented their own use of Transkribus for a set transcription project, whether personal or institutional. Bień’s study (2019) producing a digital edition of fifteenth century Polish manuscripts, accounting for their structure and special characters, is one such example. *Technological* materials were the easiest to group because of vocabulary, often discussing ways to reduce character error rates (CER) (the percentage of characters which are incorrectly recognised after training a HTR model) and utilise recurrent neural networks (RNN) (Sanchez et al. 2016). They were predominantly formatted as journal articles and conference papers. Publications in the third category, *tutorials*, usually summarised Transkribus’s capabilities and the role of READ, coming in a variety of formats including video recordings, presentations and journal articles. *User* focused materials formed the broadest category, spanning academic analysis of how participants engaged with Transkribus, to self-reported analysis; from crowdsourcing strategies motivating volunteers to general surveys. While these descriptions were broad by design, some materials evaded simple categorisation and could be placed under multiple labels. As a result, specific categorisations of approaches and methodologies are presented, below. Figure 5 shows the breakdown of the categories. The majority of materials (66.67% *n* = 254) fit into the categorisation of *humanities application*. *Technological* entries made up 25.46% (*n* = 97) of the results gathered. Only 2.89% (*n* = 11) could be classed as *tutorial*s, likely because tutorials are rarely published in academic formats, nor routinely indexed within scholarly databases. READ-COOP has released a set of comprehensive online guides to Transkribus itself (https://readcoop.eu/transkribus/resources/how-to-guides/), covering topics from downloading the software to building HTR models and producing automated transcripts.

### User research

User research appears to be underrepresented in the Transkribus literature. These materials accounted for only 4.99% (*n* = 19) of the entire dataset and 8.57% of ‘direct’ Transkribus results (*n* = 12). Most user-focused Transkribus research fits into the approach of citizen science: where researchers interact with the public to achieve a collective goal. In the case of Transkribus, this occurs with the transcription and correction of a set body of text.

Both Van Lit’s analysis (2019) of the use of Zooniverse to help crowdsourcing projects, building datasets beyond those possible using a small academic team, and Ridge’s practical guide (2020) to designing and running successful crowdsourcing projects, feature obvious citizen science applications. Other user-oriented works, mentioning Transkribus more directly than Van Lit and Ridge, discuss tranScriptorium (a precursor to the current Transkribus organisation) (Sanchez et al. 2018). TranScriptorium split users into two branches: volunteers collaborating on large projects and individual researchers using the software on their own documentation. Current attitudes among Transkribus researchers and developers continue to use this binary distinction, although papers are beginning to emerge which provide more detailed and nuanced accounts of HTR users. Chen et al. (2018) bring ideas of gamification into the discussion, using gaming dynamics in non-game settings, to entice participants to transcribe at a more productive rate.

Additionally, while the majority of research into Transkribus occurs within traditional settings, shown in Laroche’s case (2018) working with institutions like the Folger Shakespeare Library to transcribe early modern recipe books, some user studies fall outside this environment. One such study is Christlein (2018) focusing on the merits of outsourcing the transcription of early modern records from Nuremburg to naïve transcribers, those who can decipher characters without an overall language proficiency, in Vietnam due to low cost labour, using corrective technology to amend results. Subsequently, materials differ in how much trust they afford users of the platform. In Ridge’s case (2020), participants appear free to undertake as much work as they are able or want to: learning more about records as their tasks move beyond ‘business as usual’ transcription. Mirroring this, archives and libraries are seen as possible *loci* of innovation and development, with users holding a great amount of influence and insight (Chambers 2019).

**Fig. 6:**
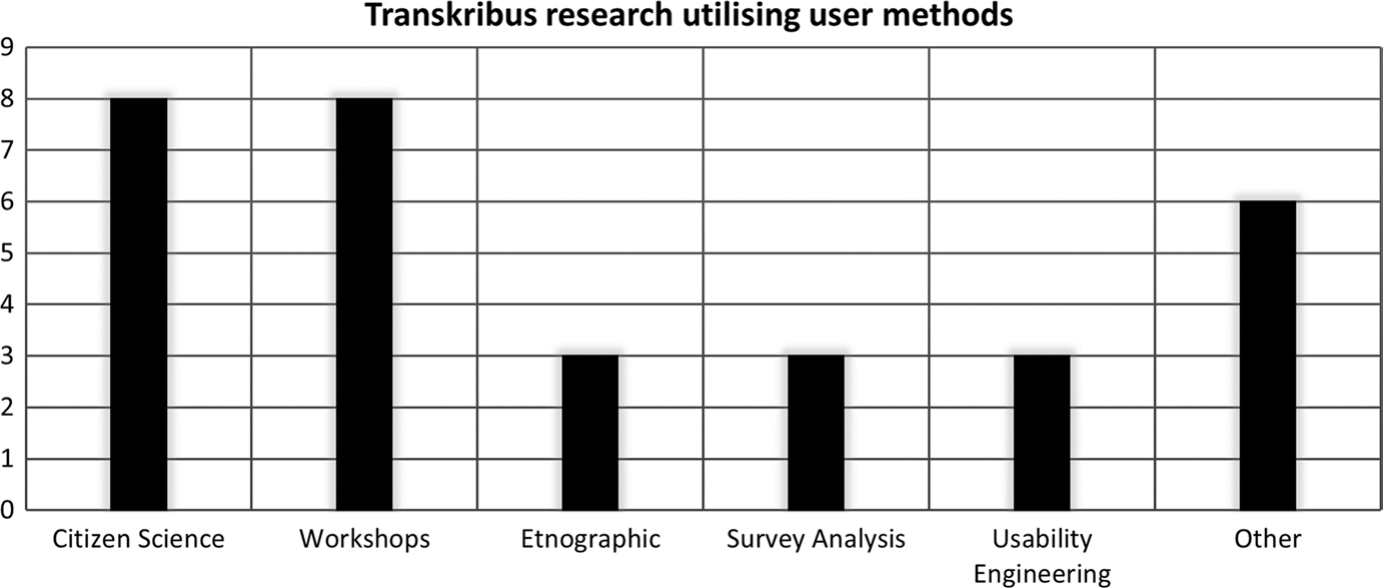
Chart highlighting Transkribus research materials which utilised user methods without falling under the category of *user*

Transkribus research which was not fully described under the category *user* but utilised similar methods provided an additional sense of the shape of the literature. Teasing out the related methodologies, various contextual frameworks can be identified in Transkribus research. While only 4.98% of works fell under the *user* classification label (*n* = 19), a greater number of results utilised user research methods. While some of these materials cannot be said to be fully user-oriented, they act as hybrid works considering many of the same issues. Approaches which were seen as falling under the bracket of user research included workshops (2.10% *n* = 8), citizen science (2.10%); ethnographic materials (0.79% *n* = 3), usability engineering (0.79%), survey analysis (0.79%) and other forms of user analysis which were harder to define, such as the opening introductions to Transkribus’s user conferences (1.57% *n* = 6). These are shown below in Fig. 6*.*

Taken alone, none of these user-led approaches to Transkribus are widely practised. This is obvious when comparing these subsets to other approaches, for example materials using musicology accounted for 1.31% of results alone (*n* = 5). Surveys of users and works from a usability engineering viewpoint are less represented than works looking at crowdsourcing methods, some of which have already been discussed. Therefore, while areas like Transkribus’s Graphic User Interface (GUI) were discussed briefly, notably in Miloni’s survey (2020) of prominent libraries using the software, a fuller review of usability for non-academic users is increasingly pressing as the platform grows. Similarly, ethnographic approaches in Transkribus research, where the researcher studied those carrying out tasks using the platform for example through ‘think aloud protocols’ (having participants vocalise their difficulties and successes) were relatively under-utilised. Gaps in user researchare clear. That said, the embryonic diversification of user analysis occurring in Transkribus research should form a useful resource for archives and libraries wanting to better understand how to embed HTR software into their accessible resources.

**Fig. 7:**
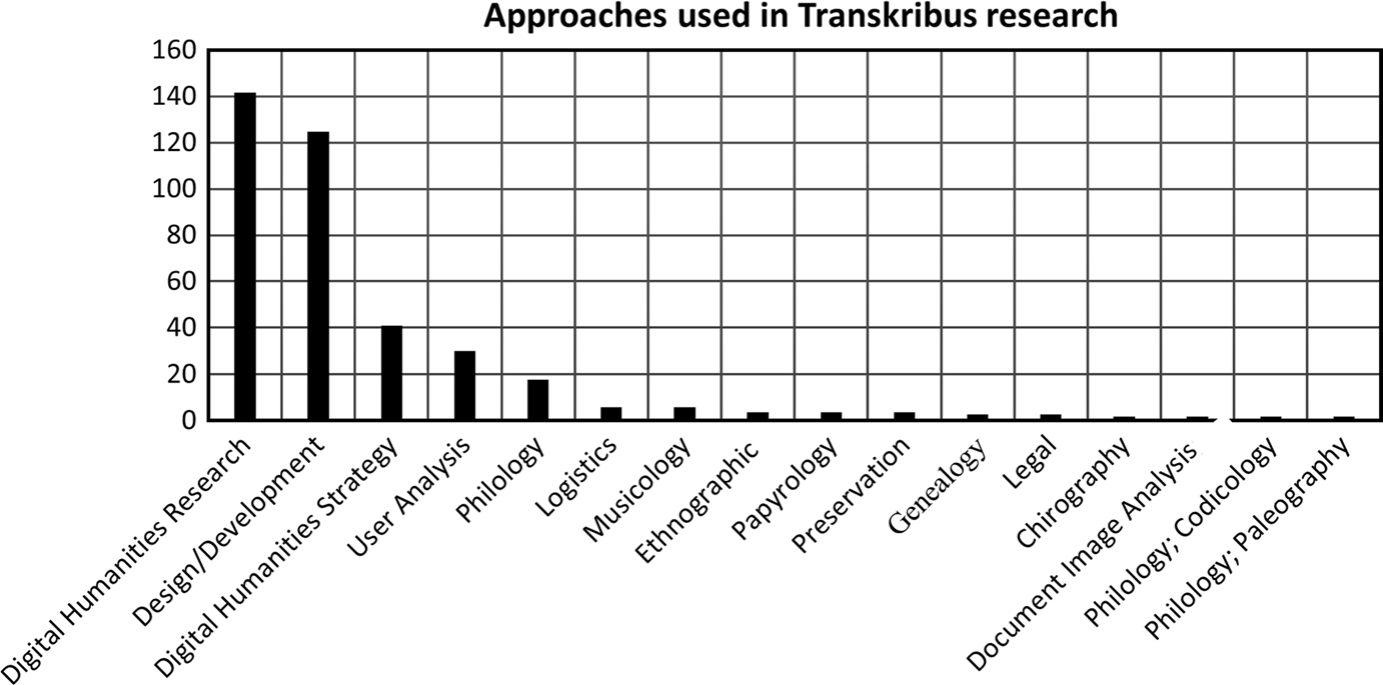
Chart of Transkribus materials by research approach and methodology. In the case of the small number of materials which used mixed methods explicitly and could not be determined to have a singular main approach, such as the result shown as philology; codicology (*n* = 1), a separate column was made. Approaches shown in Fig. 6 have been left out

Despite the low percentage of materials which are user-oriented, personal reflections on using Transkribus are often the first step in studying the HTR application. Works defined by this literature review as falling into the bracket of digital humanities research (37.01% *n* = 142) usually met this criteria, as Fig. 7 demonstrates. After this reflective statement, publications gravitate towards the technical implications of Transkribus and how its specifications could be improved upon. An example of this is Grüning’s research (2018) into baseline detection in archival documents with differing page layouts and degradation levels that challenge normal segmentation methods. However, in the wake of this interest, user analysis remains the third and final step of this evolution in research. While it is certainly the case that papers on *humanities application* and *technological* topics remain important, the lack of formal user research is problematic because it reflects a wider problem with a lack of user studies, and associated metrics for analysing and comparing usage across digital collections (OCLC Research 2015). If the community is able to address this lack of user research in relation to HTR, it would go a long way in understanding how best to serve the needs of a diverse range of users, while also recording changing methods and approaches.

The transition between the loosely defined stages of Transkribus research can be seen in the trajectory of works by lead authors. For instance, Romein’s initial work (2019) into sixteenth- and seventeenth-century legal texts from Flanders and Holland, began with a personal reflection of using Transkribus. This turned into contributing on collective projects concerning how useful the software would be on public ordinances within a similar date range and region (2019). Romein’s research has now begun to consider the changes text recognition may have on the publication and dissemination of information (2020), a focus which begins to border on what this review has described as *user* research. To date, the focus of these papers appears to have been more theoretical and critical in nature, with relatively few empirical studies of users’ experiences with Transkribus. While the former are useful, there is a need for empirical user research to enrich debates around usage. Reviews of the literature should occur regularly in order to track this progress and encourage research that enhances our understanding of the adoption and impact of HTR upon scholarly information behaviour.

### HTR and research in the humanities

Many other approaches and methodologies were catalogued during the review process, as shown in Fig. 7. Some of the more prominent approaches have already been mentioned, for example materials considering the design and development of Transkribus (32.55% *n* = 124), and those using digital humanities research methods. Alongside these, some approaches are represented far less commonly. Genealogical works, like Malmi, Gionis and Solin’s (2018) study of computer automated methods of tracking genealogical networks, were slim in number (0.52% *n* = 2). This is despite the case for using such approaches to Transkribus, uncovering personal histories and ancestral connections, being clear. Papyrology, the study of ancient handwriting on portable, often fragmentary, media, mainly papyrus, as a means of recording, analysing and interpreting text (Meeks 2020), was also present in the literature (0.79% *n* = 3). This includes examples such as Sagar’s study (2019) of OCR efficacy on palm leaves. Articles explicitly mentioning chirography, the study of penmanship and handwriting, could only be seen in 0.26% (*n* = 1) of results. Examples included Prell’s workshops (2018) on the writing practices and self-testimonies of early modern women. Although more numerous, texts approaching materials in a philological way (looking at the structure, development, and relationships of language) also formed a small portion of the whole dataset (4.99% *n* = 19). In the corpus of 140 direct works, no papers mentioned using philology outright. Of course, knowing the full extent of an approach like philology in the Transkribus body of research is difficult when compared to chirography, which is easier to catalogue due to the subject matter of handwriting being better defined.

### Technological research

92 works from the *technological* category in this review were focused on the design and development of Transkribus (*n* = 92). Interestingly, the amount of works on the design and development side of the HTR appear to be plateauing, caused, in part, by the EU funding of Transkribus ceasing in 2019. A notable increase in materials can be detected between 2017 and 2018 (*n* = 17, *n* = 26), but this upward streak began to slow in 2019, with only a slight increase from 26 to 29 entries being recorded. 2020 returned only 11 design and development materials, explained in part by the data collection process ending in October of 2020. Through this microanalytical framework, differences in tonal vocabulary were easily highlighted, and there is a tendency for technological works to consistently mention error rates for instance.

**Fig. 8:**
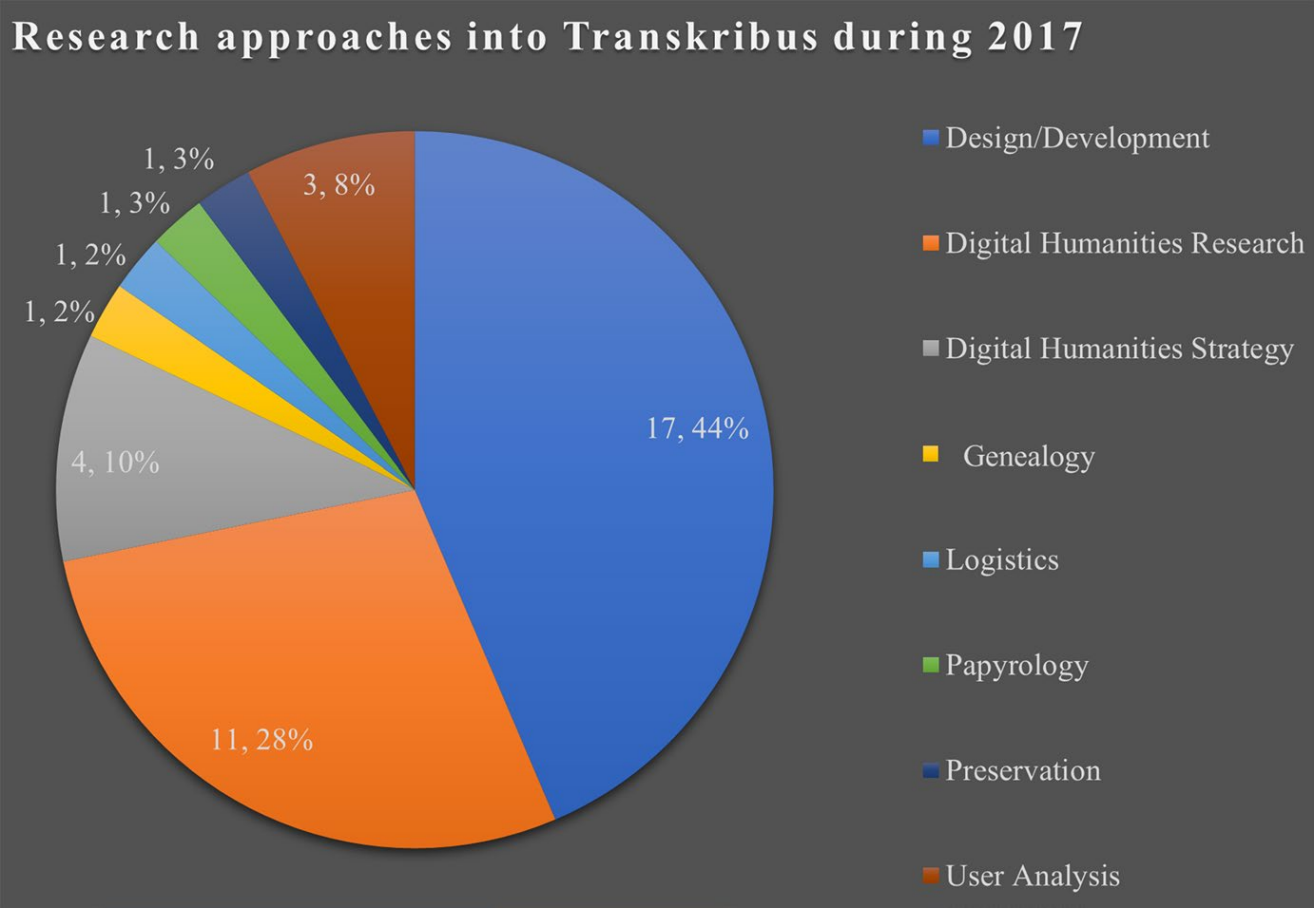
Chart showing the research approaches of 2017 Transkribus research

**Fig. 9:**
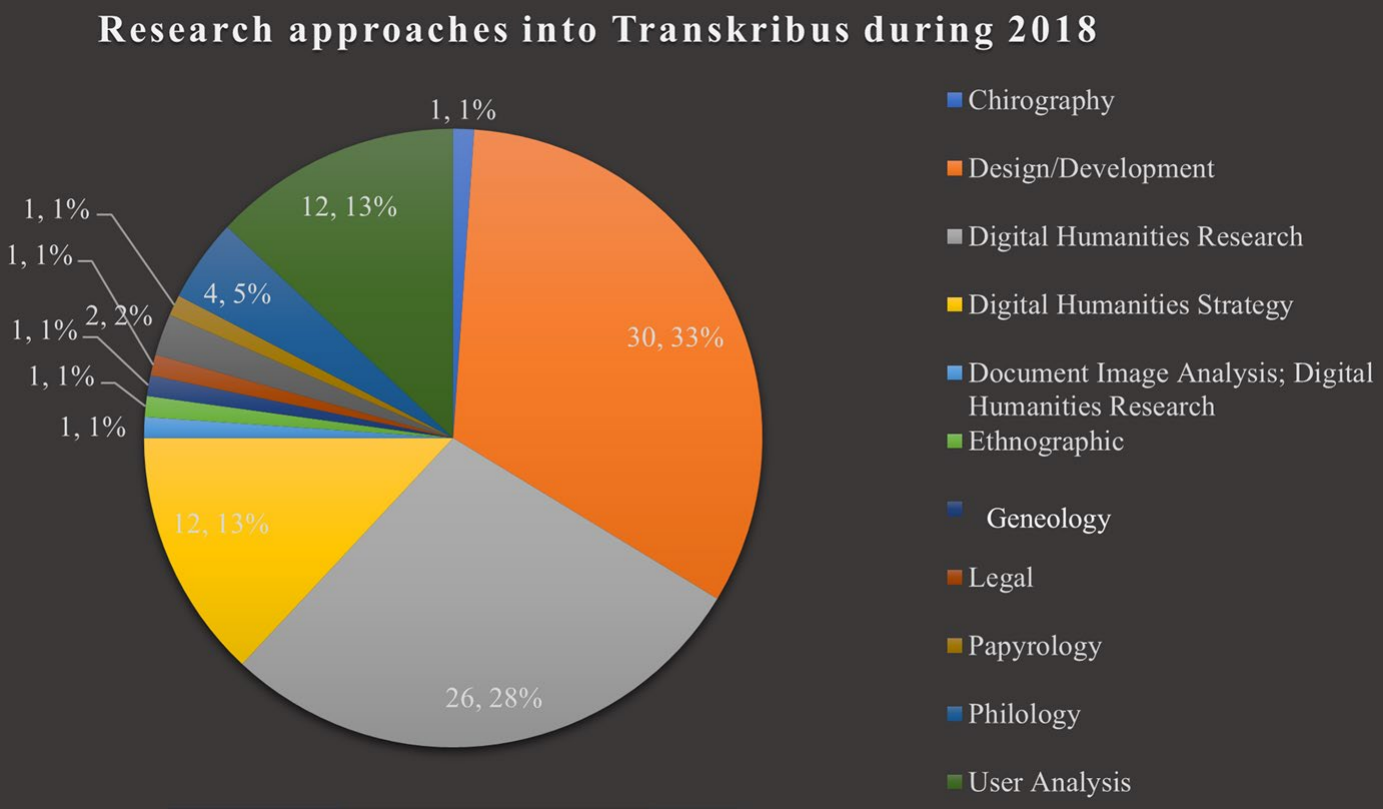
Chart showing the research approaches of 2018 Transkribus research

Figures 8 and 9 show two charts, broken down by research approach: the first from 2017 and the second from 2018. What is notable is that design and development materials (the blue segment in the first chart and the orange in the second)—while still making up a significant portion of research—appear to be receding as more work using Transkribus is conducted from a greater variety of vantage points. The case is similar if we take only the materials directly concerned with Transkribus. Between 2017 and 2018, little change occurred, the portion of research which was design and development-oriented remained high at 43.59% and 32.61%, respectively. Interest paid to such technological analysis appeared relatively constant. Yet, this changed in 2019. Only 24% (*n* = 6) of direct Transkribus materials published that year could be catalogued as design and development results, a significant drop from the previous year. Therefore, although this plateauing effect took hold of materials directly focused on Transkribus early on, in comparison to the whole corpus of literature gathered, the fall in technological interest appears much sharper.

**Fig. 10:**
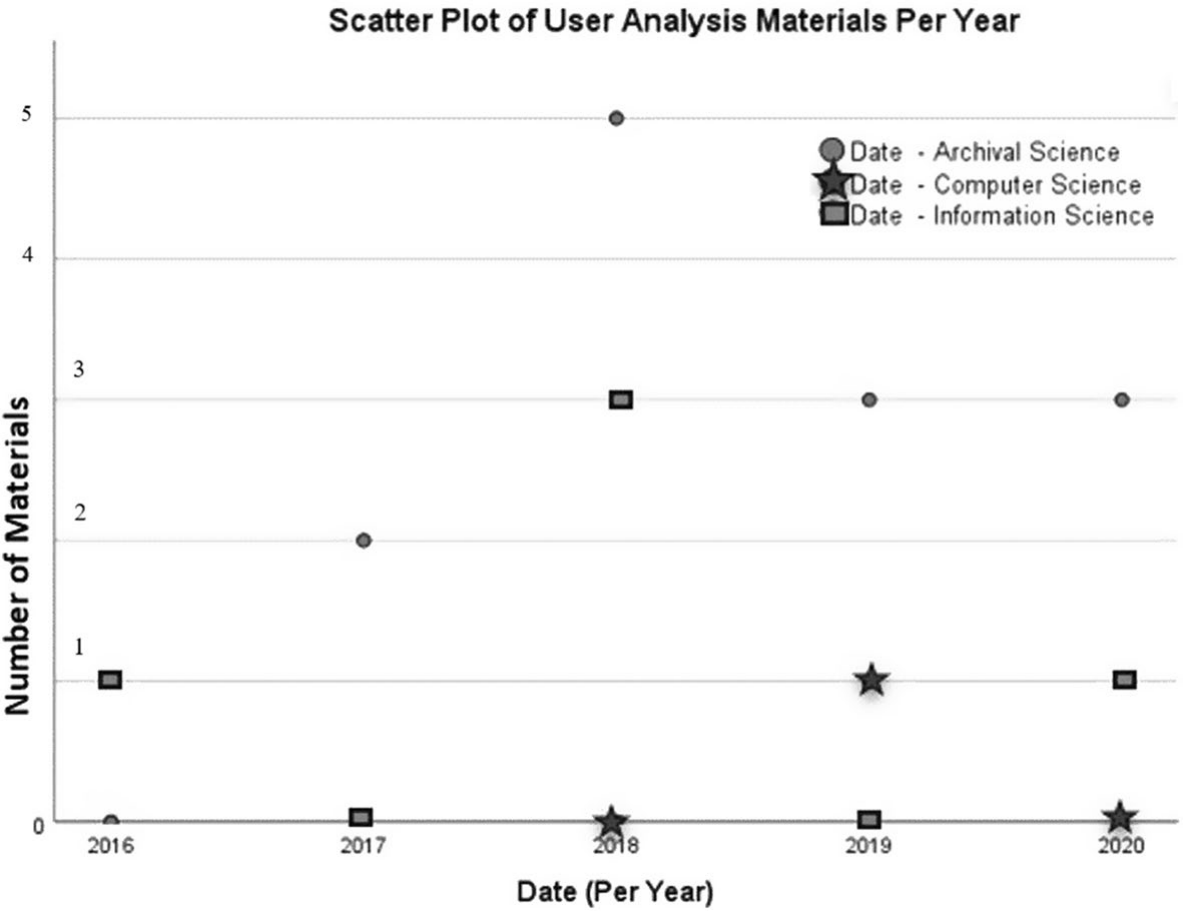
Chart of Transkribus user materials from archival science; computer science and information science (2016–2020)

Unlike the plateauing effect experienced by the more technological entries gathered and indexed for this review, user analysis materials appear to be on the rise as a proportion of Transkribus research. Again, charting the three main domains: *user* materials evidently make up a greater portion of research year on year. Works which see users as of evidential value appear most frequently among gathered archival science materials, with 2016 being the only year shown in Fig. 10 below without any such results. Information science entries fluctuate much more, with the amount of user analysis being undertaken within the domain shifting considerably from 2016 to 2020. The scatter plots from computer science serve as a hesitant reminder that we should not simply conclude that user research is on the rise without considering that Transkribus materials are still heavily weighted towards the technological side of the platform and its design and development. It is natural that computer science researchers would engage more in technological developments. As such, this stratification indicates the ways different fields engage with HTR in relation to their own domains.

While the design and development classification included the data of 92 results, Fig. 10 of *user* materials visualises only that of 17 materials. Any corrections to address this balance may be slow to be enacted, especially when research on the technological areas of Transkribus appear to be constant. Nevertheless, work on user communities has grown gradually in each year Transkribus has being researched.

### Research domains

One final element of the Transkribus literature, which is yet to be fully considered, is the full range of research domains covered in this review. It should come as no revelation that Transkribus is being used by multiple communities for multiple purposes. We have already discussed archival and library science, information science and computer science (Robinson 2009). Each field conceptualises using data and data-driven tools differently. A librarian may use HTR not to produce full transcriptions but to keyword spot metadata across collections, focusing on preparing cultural heritage collections for computational forms of research and teaching, ‘… producing data that references the same vocabularies and thesauri …’ between holdings (Lincoln 2017, p 30). This use of Transkribus could improve access to historical material for large communities of users. In contrast, individual researchers tend to use HTR software to produce rich data that is ‘replete with enough specifics that they may operationalize that data in pursuit of their research goals’ (Lincoln 2017, p 30). In this case, HTR supplements the palaeographical skill of the researcher, allowing them to draw conclusions in less time than they normally could. In terms of the domain of computer science, research into Transkribus is more dependent on gaining predictable and regularised results (such as the error rates incurred through document layout analysis techniques).

Due to the plethora of ways a tool like Transkribus is used, often within an interdisciplinary environment, categorising research domains is challenging. Therefore, how researchers described their own work formed a major consideration. Burghardt’s description of their work (2018), using optical music recognition and retrieval methods to discover melodic similarities between historic tunes, proved useful in reaching the domain of musicology. The field of history (18.37% *n* = 70) was a difficult categorisation too, as various subfields emerged, for instance labour history (1.31%, *n* = 5) or colonial history (0.79%, *n* = 3). An example of this is Prell’s study (2018) of early modern female writing practice, which begins with a description of their position as chair of the University of Jena’s Historical Institute of Gender History, making the categorization ‘gender history’ apposite. Nonetheless, Prell’s research shared similarities with all historical research as a ‘… bringer of order to the past …’ (Anderson 2004, p 82). Therefore, these subfields were folded into the more general domain of history, as atomising the historical research field at the expense of others created imbalance. The field of history also proved difficult to categorise as certain subfields of the discipline remain disputed. 48 articles (12.59%) could have been described, through a close reading of their keywords, titles and abstracts, as forming digital histories: best understood as an approach to examine and represent the past with new communication technologies, harnessing hypetextual power to define, make, query and annotate associations in the human record. With the ubiquity of the internet, some suggest that there will soon be no such distinct field (Seefeldt and Thomas III 2009; Romein et al 2020a, b).

Other articles also proved harder to contain within a domain label, such as Bonhomme’s study (2019) of how to make Parisian notaries accessible throughout automated handwriting tools at the French National Archives. Despite researching, analysing, and interpreting the past to extract meaning and establish patterns like Prell (2018), this work eventually sat between ‘history’ and archival science’ based on the content of the title, keywords and abstracts. To provide confidence in our coding, labels chosen were checked by multiple researchers to ensure agreement, as is standard practise in content analysis (Krippendorff 2004) and allied grounded theory research (Corbin and Strauss 2008).

**Fig. 11:**
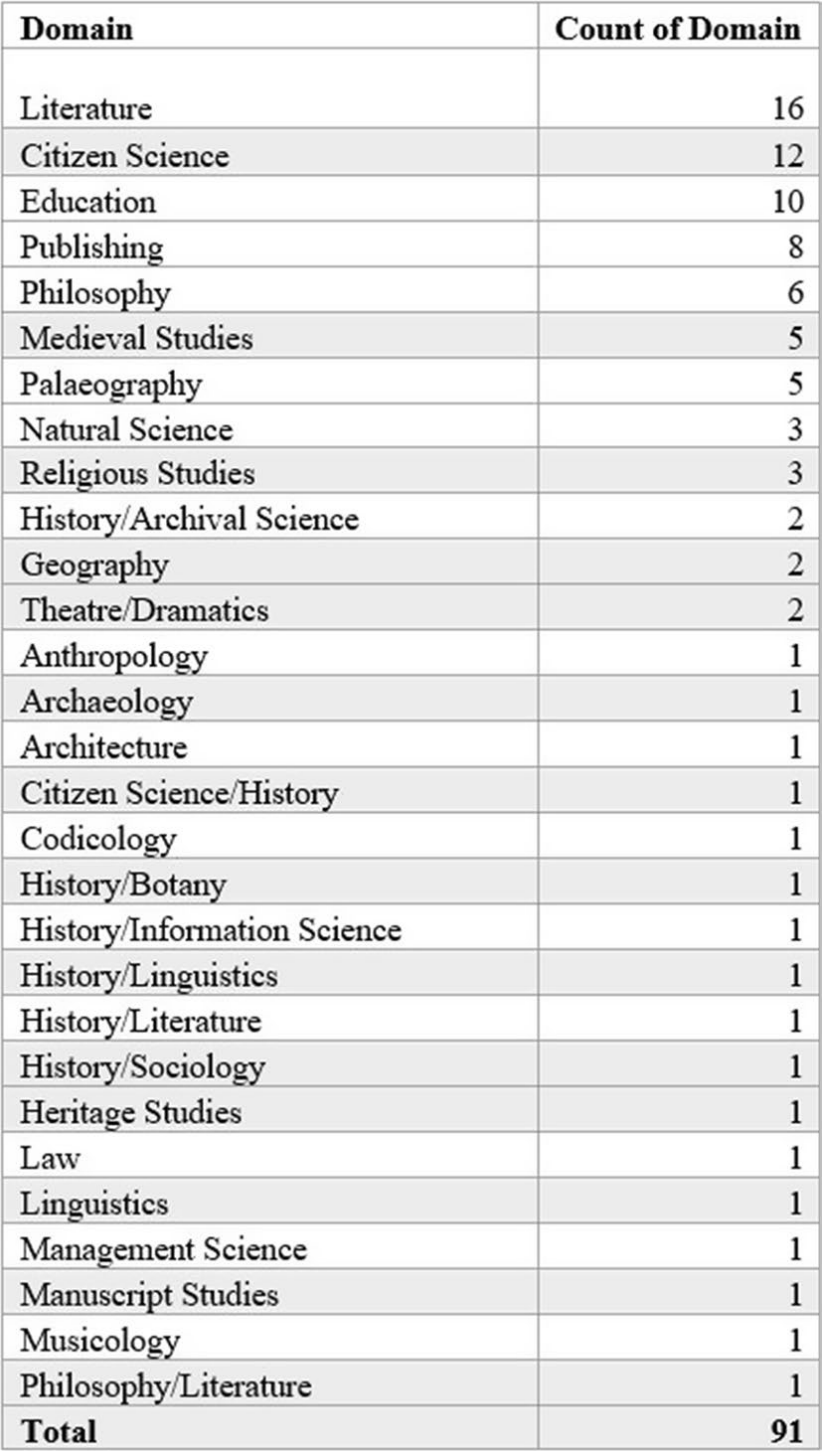
Table showing all domains accounting for less than 10% of the total items indexed (2016–2020)

It is worth mentioning those domains which accounted for less than 10% (*n* = 38) of the total research into the HTR platform. Although small in number, these materials show the true span of how Transkribus has been used in the research community: the results of which are shown in Fig. 11. Ranging from being utilised on legal texts to charting linguistical terms over time, the ways Transkribus has been used highlight one of the main advantages of the platform—that it appears omnivorous in the documentation it can transcribe and robust in dealing with a variety of scripts (depending on the skill of the transcriber and quality of the image to be processed).

**Fig. 12:**
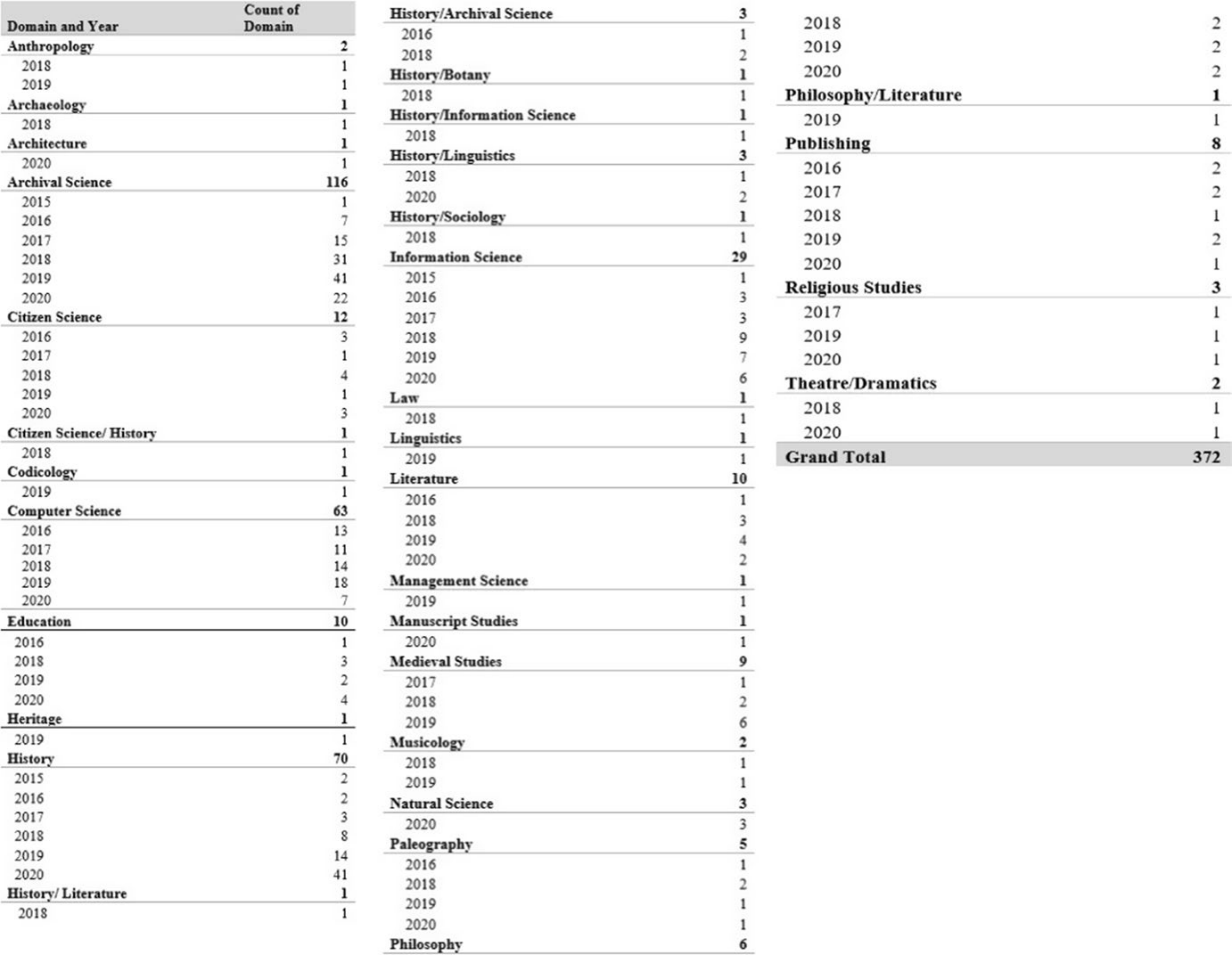
Table showing the number of papers from each research field in the Transkribus corpus by year

Of results indexed, 2.62% could be categorised as within the field of education, moving past teaching Transkribus to potential users in the form of tutorials to considering how best to frame the digital humanities to new students (Kaden and Kleineberg 2019). Even materials from the domain of theatre and dramatics, architecture and botany were listed and included in this review. In the case of the theatre entry, the material came in the form of a production drawing on the correspondence of two Germans written using transcription tools (Baker et al. 2017). Overall, 31 domains were listed from the total dataset of 381 works. If we pick out the range of domains per year of Transkribus research, we can see a clear diversification of study using the text recognition platform after 2017. 33 items were indexed from 2016, representing 10 domains. In 2017, the items returned increased marginally to 37 but the range of domains dropped to 8. This changed dramatically in 2018 with an increase in materials (*n* = 98) and domains (*n* = 22). While Transkribus research items increased in 2019 (*n* = 100), the domains seen were fewer but still much higher than they had been in 2017 and 2016 (*n* = 16). This continued into 2020 with an equal number of materials (*n* = 99) and fewer domains (*n* = 16) accounted for but remaining higher than pre-2018 levels. In 2016, works from archival and library science dominated research, with education and history the only other fields present. Since 2017, research into Transkribus has been undertaken in a variety of fields. While archival science, information science, and computer science remain the dominant fields, we found that work has been published across the arts, humanities and social sciences. Fields that were represented in our corpus included religious studies, publishing, history, theatre studies, philosophy, management science, and medieval studies. Figure 12 displays the range of research fields found by year with the corresponding number of papers indexed.

How deeply ingrained the use of Transkribus may become in these research fields is hard to ascertain but it remains clear that research into HTR has diversified in recent years, benefiting a greater amount of researchers through emerging intersectional links.

## Discussion

Transkribus was the only free consumer-level HTR at its conception, recently changing to a pay-for model. How this change impacts the use and expansion of the platform will need to be studied in the future. The 31 domains accounted for in this study of Transkribus materials ranged from archival science and computer science to the plethora of fields making up less than 10% of the works indexed. This creates a firm impression that Transkribus is both omnivorous in its intake and useful to a range of researchers working on different kinds of material. With the development of the software moving to smaller details, as shown in Figs. 8 and 9, research using and into Transkribus is beginning to enter a new stage: applying the software on an increasing range of documentation using new methodologies. It is too early to judge whether the recent change to a paid credit-based model, necessitated by the end of EU funding for Transkribus in 2019, will inhibit this growth. The essence of Transkribus as a ‘bottom-up’ mass digitization movement, made up of hundreds of simultaneous projects driven by motivated researchers, gives the best chance of success for the READ-COOP (Thylstrup 2019; Benoit and Eveleigh 2019), due to the platform’s reliance on ground truth data being gathered from multiple users transcriptions and the exchanging of models. As Transkribus grows, more research will inevitably be produced and new rhythms will emerge in the approaches of researchers. Carrying out subsequent systematic analysis of the literature will allow this to be tracked and understood.

## Conclusion

This review has catalogued a corpus of publications gathered by searching for ‘Transkribus’ in Google Scholar, Scopus and Web of Science. It has served to show the current shape of research into and using Transkribus. By gathering and categorising publications from different scholarly databases, we were able to identify 381 outputs that mentioned Transkribus to some degree. In systematically analysing these, we provide a snapshot of the current use of HTR by researchers: mostly in the cultural heritage domain.

Published research on Transkribus is undergoing a steady rise, and a main finding of this research is that these studies—and by implication HTR—are broad and eclectic. This is especially clear when considering the bottom-up and cooperative structure of the platform, fostering the collaborative use and development of recognition models. Transkribus research shows no signs of becoming more homogenous but instead is reaching into new domains such as botany and architecture. This is a reflection on the software, which appears domain-agnostic, meeting various user requirements, from producing general data charting similarities across collections to producing findings replete with specifics.

Content analysis allowed the development of taxonomical classifications for the indexed materials, using the labels *humanities application*, *technological*, *user* and *tutorial*. These categories provided a sense of the latent context of materials gathered and helped in the data sorting and cleaning of results. This meant that even texts with limited descriptive data could be categorised. For those wanting to access an authoritative bibliography of research into HTR, we have published the resulting full list of papers as a downloadable appendix on Zenodo.

What research rhythms will emerge from utilising Transkribus in the future is uncertain, as is whether the list of fields using the platform will continue to diversify. It is a reasonable assertion, due to archives engaging with digitisation *en masse*, especially since the Covid-19 pandemic, and user communities beginning to prefer accessing digital surrogates of materials online (Chassanoff 2013), that a rise in the number of published materials mentioning Transkribus may soon replicate the 235% jump from 2017 to 2018. In addition, it is possible that user focused analysis will continue to increase, as it has done since 2016. This is subject to our previous observation that it is not yet clear whether the increased profile of critical and self-reflective commentary on Transkribus usage will be followed by empirical user analysis which models scholarly information behaviours when using HTR.

Written within an increasingly saturated space, materials tackling the design and development of Transkribus may decrease now the HTR software has achieved greater maturation (coinciding with the grant-funded development of the platform ceasing, lessening development time and imperatives to publish results). Likewise, we should watch carefully whether Transkribus research will retreat from formalised academic publication. As stated, 42.78% (*n* = 163) of materials included in this literature review came in the format of journal articles. Nevertheless, as the use of Transkribus branches out beyond traditional settings and institutions, shown in Christlein’s work (2018) on employing naïve transcribers through private companies, research into and using the platform may appear in a greater variety of formats and spaces. Work using network analysis, uncovering patterns underneath empirical observations about Transkribus research, may soon be needed to monitor these changes. One method could be egocentric network analysis, where scholars of Transkribus report their research domain and what approaches they have been using (possibly through a READ form or compiled as part of a public database). From then, paths and geodesic values (measuring the shortest distances between certain characteristics of the body of research) could be established (Knoke and Yang 2011). After gaining consent, this method of self-reporting could be useful for future surveys. Through this, answers concerning whether the pay-for model has impacted Transkribus’s use could be reached. Such a method, as opposed to other resuggestions like link analysis, could avoid a metric-driven account of the literature and give user communities a clearer voice in defining the themes and direction of HTR research (Gooding 2018).

In this systematic literature review, we aimed to identify the key domains and essence of Transkribus research, while showing that HTR is now being used by a broad set of research domains. HTR’s growing interest is likely to continue, partly due to the ongoing Covid-19 pandemic, which has inadvertently provided a window for archives and libraries to prioritise digital projects, delivering services through multiple channels for those without access to buildings (National Library of Scotland 2020). HTR has also grown in use through word-of-mouth among the research community as a result of its accuracy. These matters will need to be monitored through open forums among Transkribus users and through subsequent literature reviews, with administrators regularly updating the research methods, much like the initiatives taken after the report of digital editing work by Franzini et al. (2016). Through such a structure, the greater recognition of the evidential value of user experiences with HTR could be reached, developing the technology in a sustainable and useful manner. In doing so, important evidence will emerge on how HTR infrastructure can be built to support broader research communities.

## Data Availability

A list of all papers found will be provided as a downloadable appendix.
